# p53 isoform Δ113p53 promotes zebrafish heart regeneration by maintaining redox homeostasis

**DOI:** 10.1038/s41419-020-02781-7

**Published:** 2020-07-23

**Authors:** Shengfan Ye, Ting Zhao, Wei Zhang, Zimu Tang, Ce Gao, Zhipeng Ma, Jing-Wei Xiong, Jinrong Peng, Wei-Qiang Tan, Jun Chen

**Affiliations:** 1 MOE Key Laboratory of Biosystems Homeostasis & Protection and Innovation Center for Cell Signaling Network, College of Life Sciences, Zhejiang University, 310058 Hangzhou, China; 2 College of Animal Sciences, Zhejiang University, 310058 Hangzhou, China; 3 Institute of Molecular Medicine, Beijing Key Laboratory of Cardiometabolic Molecular Medicine, and State Key Laboratory of Natural and Biomimetic Drugs, Peking University, 100871 Beijing, China; 4 Department of Plastic Surgery, Sir Run Run Shaw Hospital, Zhejiang University School of Medicine, No. 3 Qingchun Road East, 310016 Hangzhou, China

**Keywords:** Cell proliferation, Reprogramming

## Abstract

Neonatal mice and adult zebrafish can fully regenerate their hearts through proliferation of pre-existing cardiomyocytes. Previous studies have revealed that p53 signalling is activated during cardiac regeneration in neonatal mice and that hydrogen peroxide (H_2_O_2_) generated near the wound site acts as a novel signal to promote zebrafish heart regeneration. We recently demonstrated that the expression of the p53 isoform *Δ133p53* is highly induced upon stimulation by low-level reactive oxygen species (ROS) and that Δ133p53 coordinates with full-length p53 to promote cell survival by enhancing the expression of antioxidant genes. However, the function of p53 signalling in heart regeneration remains uncharacterised. Here, we found that the expression of *Δ113p53* is activated in cardiomyocytes at the resection site in the zebrafish heart in a full-length p53- and ROS signalling-dependent manner. Cell lineage tracing showed that *Δ113p53*-positive cardiomyocytes undergo cell proliferation and contribute to myocardial regeneration. More importantly, heart regeneration is impaired in *Δ113p53*^*M/M*^ mutant zebrafish. Depletion of *Δ113p53* significantly decreases the proliferation frequency of cardiomyocytes but has little effect on the activation of *gata4*-positive cells, their migration to the edge of the wound site, or apoptotic activity. Live imaging of intact hearts showed that induction of H_2_O_2_ at the resection site is significantly higher in *Δ113p53*^*M/M*^ mutants than in wild-type zebrafish, which may be the result of reduced induction of antioxidant genes in *Δ113p53*^*M/M*^ mutants. Our findings demonstrate that induction of *Δ113p53* in cardiomyocytes at the resection site functions to promote heart regeneration by increasing the expression of antioxidant genes to maintain redox homeostasis.

## Introduction

The adult mammalian heart has limited regenerative capability following cardiac damage, and this is the main reason that cardiac infarction is one of the leading causes of death worldwide^1^. In contrast, the hearts of adult zebrafish and neonatal mice exhibit full cardiac regeneration capacity following ventricular resection or cryoinjury through robust cardiomyocyte proliferation^2–4^. In zebrafish, cardiomyocytes from the subepicardial ventricular layer dedifferentiate into *gata4*-positive cardiomyocytes to proliferate and invade the area of injury, and this is the major process underlying heart regeneration^5,6^.

A number of signalling pathways, including the Notch, BMP, PDGF, RA, Nrg1 and Brg1 pathways, have been documented to regulate zebrafish cardiac regeneration^7–15^. Reactive oxygen species (ROS), specifically H_2_O_2_, produced in the epicardium and adjacent myocardium near the wound site have also been found to promote the proliferation of cardiomyocytes^16^. ROS, including superoxide anion (O_2_^•–^), hydroxyl radical (OH^•^) and the non-radical species hydrogen peroxide (H_2_O_2_), play a dual role in cell fate determination. At moderate levels, ROS can function as signals that promote cell growth and division^17–19^. In contrast, when ROS are overproduced beyond a cell’s capacity to maintain redox homeostasis, they can lead to oxidation of macromolecules such as proteins, membrane lipids and mitochondria and genomic DNA^20,21^. The harmful accumulation of ROS eventually results in abnormal cell death and senescence.

To maintain redox homeostasis, organisms have evolutionarily developed numerous antioxidant defence systems, including both enzymatic and non-enzymatic antioxidant mechanisms that can either scavenge ROS or prevent their formation^22^. In response to oxidative stress, the signalling pathway of the tumour repressor p53 plays important and complex roles^23–26^. Under physiological conditions and during low levels of oxidative stress, p53 functions to maintain oxidative homeostasis and promote cell survival through transcriptionally expressing antioxidant genes^27–32^. However, p53 triggers apoptotic activity by upregulating the expression of pro-oxidative genes and apoptotic genes in response to high levels of oxidative stress^30,33–35^. Zebrafish Δ113p53 and its human counterpart Δ133p53, N-terminal truncated isoforms of p53, are both transcribed by an alternative *p53* promoter in intron 4^36,37^. Full-length p53 can directly transactivate the transcription of these isoforms in response to both developmental and DNA damage stresses^38–40^. In turn, the induction of Δ113p53/Δ133p53 inhibits p53-dependent apoptosis by differentially modulating the expression of p53 target genes^36,37,40^. Δ113p53/Δ133p53 can form a complex with p53 both in vitro and in vivo, and this interaction is essential for its anti-apoptotic activity^41^. The basal expression of Δ133p53 prevents normal human fibroblasts, T-lymphocytes and astrocytes from p53-mediated replicative senescence by repressing *miR-34a* expression^42,43^. In response to γ-irradiation, Δ113p53/Δ133p53 not only represses cell apoptosis but also coordinates with p73 to promote DNA DSB repair by upregulating the transcription of repair genes^44,45^. Interestingly, our recent study revealed that upon treatment with sub-toxic ROS stresses, Δ133p53 does not antagonise the activity of p53 but coordinates with p53 to promote cell survival by promoting antioxidant gene expression^46^.

A study in mice showed that p53 signalling is activated in cardiomyocytes during neonatal mouse heart regeneration^47^. However, the roles p53 signalling plays and whether its isoforms are activated in heart regeneration are unknown. In this report, we reveal that *Δ113p53* is induced in cardiomyocytes at the resection site in the zebrafish heart and that this induction is dependent on full-length p53 and ROS signalling. Furthermore, Δ113p53 promotes heart regeneration through upregulating the expression of antioxidant genes. Our results demonstrate that activation of the p53 signalling pathway is required for heart regeneration by maintaining redox homeostasis.

## Results

### The expression of *Δ113p53* is induced in cardiomyocytes at the resection site in the zebrafish heart

**Fig. 1:**
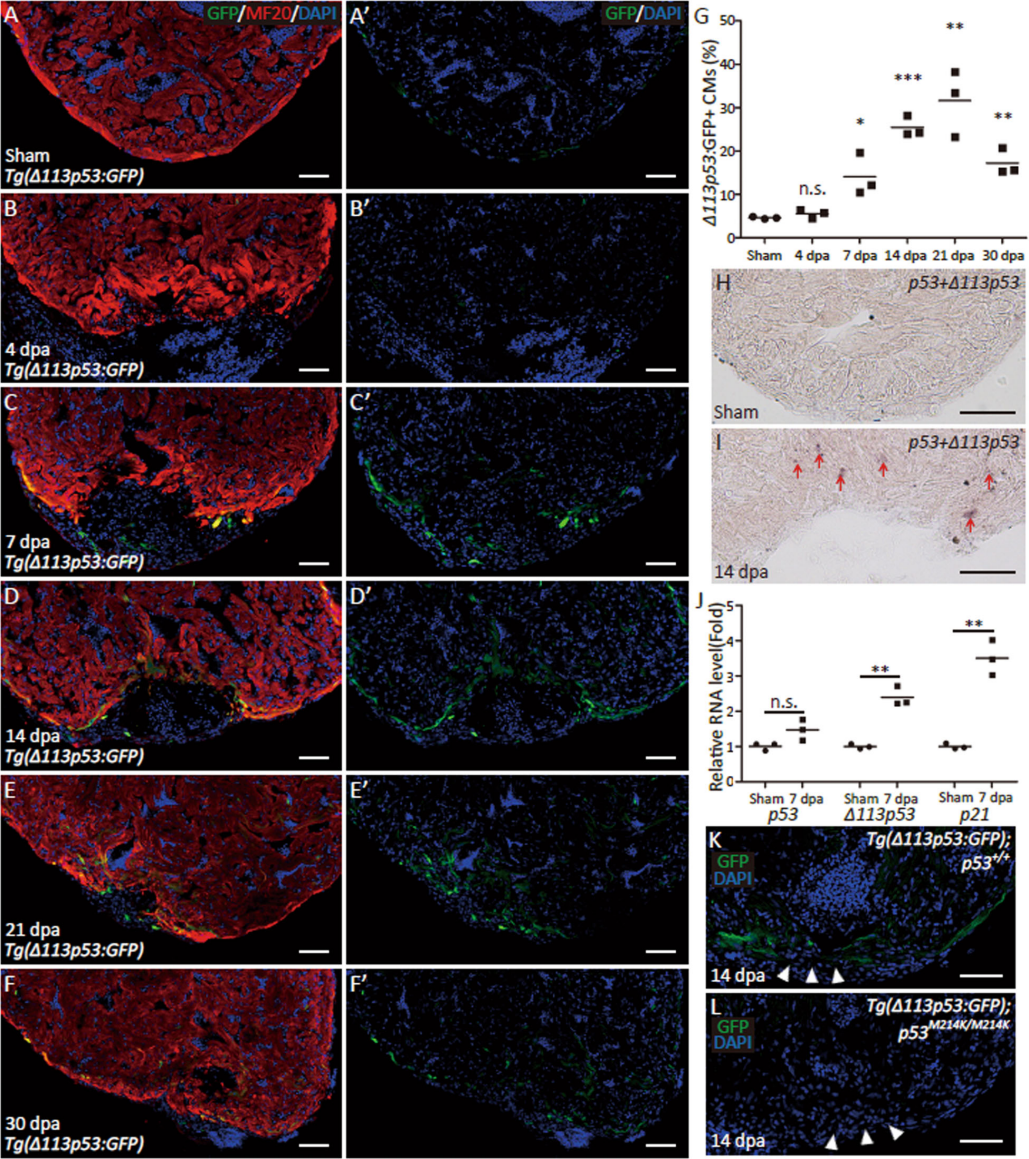
The expression of *Δ113p53* is induced in cardiomyocytes at the resection site of zebrafish heart. **a**–**f** Cryosections of *Tg(Δ113p53:GFP)* hearts at sham (**a**, **a**′), 4 (**b**, **b**′), 7(**c**, **c**′), 14 (**d**, **d**′), 21 (**e**, **e**′) and 30dpa **(f**, **f**′**)** were immunostained by anti-GFP (in green) and anti-MHC (MF20) (in red) antibodies. The nucleus were stained with DAPI (in blue). Scale bar, 50μm. **g** Average size of GFP^+^ cardiomyocytes on heart sections of *Tg(Δ113p53:GFP)* at sham, 4, 7, 14, 21 and 30dpa, was presented as the percentage of the total ventricular area. Each dot presented an individual heart. Data are means of 3 sections/heart from 3 hearts/time point. **h**, **i** RNA in situ hybridisation was performed with the DIG-labelled probe to detect both *p53* and *Δ113p53* on cryosections of WT hearts at sham (**h**) and 14dpa (**i**). The representative picture was taken from three hearts in each group. Scale bar, 50μm. **j** Relative mRNA expression of *p53, Δ113p53* and *p21* in the WT injury hearts at sham and 7dpa. The total RNA was extracted from a pool of at least 10 hearts in each group. **k**, **l** Cryosections of *Tg(Δ113p53:GFP)* hearts of *p53*^*+/+*^ sibling (**k**) and *p53*^*M214K*^ mutant (**l**) at 14dpa were immunostained by anti-GFP antibody. The representative picture was taken from three hearts in each group. The white arrow heads indicate wounding site. Scale bar, 50μm. The experiments were repeated independently for at least three times with similar results. Statistical analysis was performed on relevant data using Student’s two-tailed *t* test in GraphPad Prism 5. The *p* values were represented by n.s. and asterisks. n.s., *p*>0.05. *p<0.05. **p<0.01. ***p<0.001.

To investigate whether the p53 signalling pathway is also activated during zebrafish heart regeneration as in neonatal mice, we surgically removed ~15% of ventricular cardiomyocytes from *tg(Δ113p53:GFP)* transgenic zebrafish, in which the expression of GFP faithfully mimics the transcription of endogenous *Δ113p53*^40^. Interestingly, we found that the GFP signal was co-localised with MHC (the myosin heavy chain of cardiomyocytes) at the resection site beginning 7 days post-amputation (dpa; Fig. 1c), reached a peak at 21 dpa and decreased at 30 dpa (Fig. 1d–f), whereas the green fluorescent signal was merely observed in the ventricles of both the sham hearts and the resected hearts at 4 dpa (Fig. 1a, b, g).

To confirm the activation of the p53 signalling pathway, we performed an in situ hybridisation assay with a probe that detects both full-length *p53* and *Δ113p53*. Positive signals were observed in cells near the resection site in wild-type (WT) hearts at 14 dpa (Fig. 1i) but not in the ventricles in sham hearts (Fig. 1h). Quantitative reverse transcription PCR (qRT-PCR) showed that the expression of *Δ113p53* and *p21* (also a p53 target gene), but not full-length *p53*, was significantly increased in the resected hearts compared to the sham hearts at 7 dpa (Fig. 1j).

As *Δ113p53* is a p53 target gene, we asked whether the induction of the transgene was p53-dependent. For this purpose, the *tg(Δ113p53:GFP)* transgene was crossed into the *p53*^*M214K*^ mutant background, in which the transcriptional activity of mutant p53 is lost^48^. Unlike in the resected hearts of WT fish, GFP was not detectable in the resected hearts of *p53*^*M214K*^ mutant fish at 14 dpa (Figs. S1 and 1k, l). Taken together, the results suggest that full-length p53 was post-transcriptionally activated to upregulate the expression of its downstream genes, including *Δ113p53*, during heart regeneration.

### *Δ113p53*-positive cardiomyocytes undergo cell proliferation and contribute to heart regeneration

**Fig. 2:**
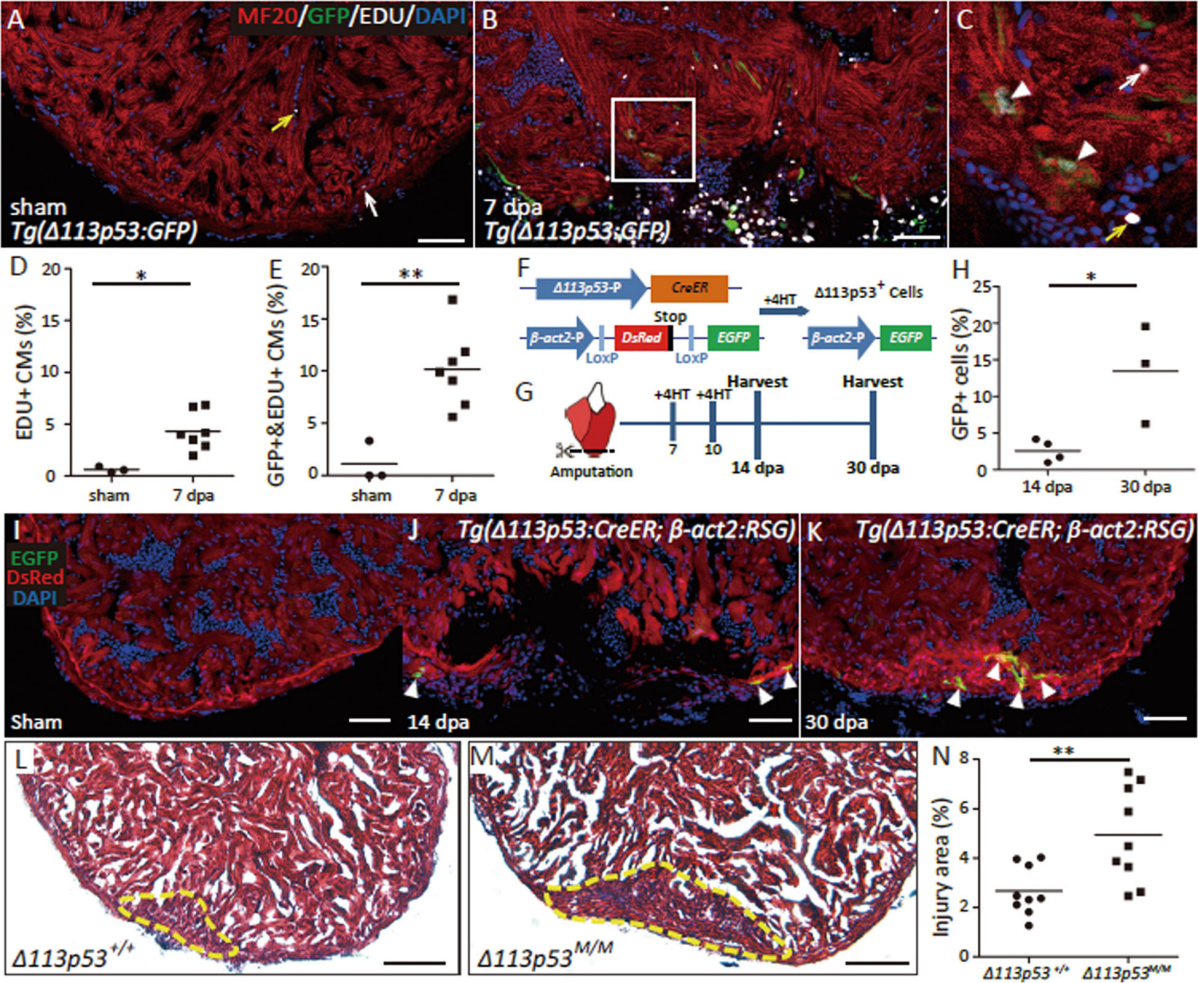
*Δ113p53*-positive cardiomyocytes undergo cell proliferation and contribute to heart regeneration. **a**–**e** Cryosections of Edu-labelled *Tg(Δ113p53:GFP)* hearts at sham (**a**) and 7 dpa (**b**, **c**) were immunostained by anti-GFP (in green) and anti-MF20 (in red) antibodies. The nucleus were stained with DAPI (in blue). Framed area in **b** was magnified in **c**. The representative picture was taken from 3 to 7 hearts. Scale bar, 50 μm. Yellow arrows: Edu^+^/GFP^−^/MF20^−^ cells; white arrows: Edu^+^/GFP^-^/MF20^+^ cells; white arrow head: Edu^+^/GFP^+^/MF20^+^ cells. The number of Edu^+^/MF20^+^ cells on heart sections of *Tg(Δ113p53:GFP)* at sham and 7 dpa, was presented as the percentage of the total MF20^+^ cells at the wound site (**d**). The number of Edu^+^/GFP^+^/MF20^+^ cells on heart sections of *Tg(Δ113p53:GFP)* at sham and 7 dpa, was presented as the percentage of the total GFP^+^/MF20^+^ cells at the wound site (**e**). Data are means of 4–6 sections/heart with the largest wound area from 3 to 7 hearts in different treatments. Scale bar, 50 μm. Each dot represents an individual heart. **f** A schematic diagram representing the 4HT-based Cre-LoxP system driven by *Δ113p53* promoter. *Δ113p53*-P (blue arrow): the 3.6-kb DNA fragment from the upstream of *Δ113p53* transcription start site; *β-act2*-P (blue arrow): the promoter of *β-actin2*; *CreER* (Brown bar): the coding region of tamoxifen-inducible Cre recombinase–oestrogen receptor fusion protein; LoxP (blue bar): the site of LoxP; DsRed (red bar): the coding region of *DsRed*; Stop (black bar): the translation stop codon; *EGFP* (light green bar): the coding region of *EGFP* gene; 4HT: the treatment of 4-hydroxytamoxifen. **g** Schematics of the cell lineage tracing experiment. Either sham or surgical *Tg(Δ113p53:CreER; β-act2:RSG)* zebrafish were treated with 4HT at 7 and 10 dpa as indicated. The treated surgical zebrafish were sampled at 14 and 30 dpa, while all of the treated sham zebrafish were sampled at 30 dpa. **h**–**k** Red and green fluorescence on the cryosections of *Tg(Δ113p53:CreErt2;β-actin:RSG)* hearts at sham (**i**), 14 (**j**) and 30 dpa (**k**) were from the en vivo DsRed and EGFP protein respectively. The nuclei were stained with DAPI (blue). Scale bar, 50 μm. The number of EGFP^+^ cells on heart sections of *Tg(Δ113p53:CreER; β-act2:RSG)* at 14 and 30 dpa, was presented as the percentage of the total DsRed^+^ cells at the resection site (**h**). Data are from the biggest section with most EGFP^+^ cells of every heart. Each dot represents an individual heart. **l**–**n** Fibrin clot stained with Masson’s trichrome on the crysections of *Δ113p53*^*+/+*^ (**l**) and *Δ113p53*^*M/M*^ mutant hearts (**m**) at 30 dpa. Yellow dotted lines indicate the approximate injury area. Scale bar, 50 μm. Average injury area with fibrin clots on sections of *Δ113p53*^*+/+*^ and *Δ113p53*^*M/M*^ mutant hearts at 30 dpa was presented as the percentage of the total ventricular area (**n**). Data are means of three sections/heart. Each dot represents the average injury area of an individual heart. The experiments were repeated independently for at least three times with similar results. Statistical analysis was performed on relevant data using Student’s two-tailed *t* test in GraphPad Prism 5. The *p* values were represented by n.s. and asterisks. n.s., *p* > 0.05. *p < 0.05. **p < 0.01. ***p < 0.001.

To explore whether the induction of *Δ113p53* is related to the proliferation of cardiomyocytes, we subjected *tg(Δ113p53:GFP)* zebrafish to EdU (5-ethynyl-2′-deoxyuridine)-labelling from 5 to 7 dpa. At 7 dpa, ~4.3% of cardiomyocytes (MF20-positive cells) in the wound area were labelled with Edu (Fig. 2b–d), whereas up to 10.2% of *Δ113p53*-positive cardiomyocytes were labelled with Edu (Fig. 2e). The Edu-labelled *Δ113p53*-positive cardiomyocytes accounted for 24.4% of total Edu-labelled cardiomyocytes (Fig. S2). The Edu-labelled cardiomyocytes or Edu-labelled *Δ113p53*-positive cardiomyocytes were rarely observed in the sham hearts (Fig. 2a). The results demonstrate that many *Δ113p53:GFP*^*+*^ cells near the lateral edges of the wound have newly undergone DNA synthesis.

To investigate the dynamics of *Δ113p53*-positive cardiomyocytes in heart regeneration, a cell lineage tracing assay was performed. We generated *tg(Δ113p53:CreER)* transgenic zebrafish using a 3.6-kb fragment of the *Δ113p53* promoter to drive CreER (tamoxifen-inducible Cre recombinase–oestrogen receptor fusion protein) expression and crossed them with *tg(β-act2:RSG)* zebrafish to generate *tg(Δ113p53:CreER*; *β-act2:RSG)* double transgenic fish (Fig. 2f). Our previous study revealed that the expression of Δ113p53 is strongly induced upon treatment with DNA-damaging drugs^40^. To verify the utility of the double transgenic fish, the transgenic embryos were treated with either camptothecin (Campt, a DNA-damaging drug), 4-hydroxytamoxifen (4HT) or a combination of both. Western blot analysis showed that the expression of endogenous Δ113p53 was induced by Campt but not by 4HT (Fig. S3a). Green fluorescence appeared in the transgenic embryos treated with the combination of Campt and 4HT (Fig. S3e) but not in the untreated embryos or the embryos treated with either drug alone (Fig. S3b–d). The results demonstrated that the double transgenic fish could be used to trace the induction of *Δ113p53*.

Next, we treated the sham zebrafish and adult double transgenic zebrafish subjected to surgery with 4HT at 7 and 10 dpa (Fig. 2g), the time points preceding detectable of *Δ113p53-*driven GFP fluorescence in the injury site. At 14 dpa, a small number of EGFP^+^ cardiomyocytes (2.6%) were detected near the border of the wound in the 4HT-treated *tg(Δ113p53:CreER*;*β-act2:RSG)* animals (Fig. 2j, h) but not in the sham controls (Fig. 2i). Moreover, the number of EGFP^+^ cardiomyocytes significantly increased to 13.5% at 30 dpa (Fig. 2k, h). These results indicate that *Δ113p53-*positive cardiomyocytes undergo cell proliferation and contribute to heart regeneration.

### Heart regeneration is impaired in *Δ113p53*^*M/M*^ mutant zebrafish

During zebrafish heart regeneration, a large clot of blood cells (most of them being erythrocytes) forms in the resection site after a few seconds of profuse bleeding from the ventricular lumen; these blood cells are replaced by fibrin beginning 2 dpa. Cardiomyocytes surround, penetrate and finally replace the fibrin clot from 9 to 30 dpa^2^. The area of the injury containing the fibrin clot is a critical parameter for evaluating the quality of heart regeneration^49^. To investigate the role of *Δ113p53* in heart regeneration, we performed Masson’s staining to compare the area of the injury containing the fibrin clot between the resected hearts of WT zebrafish and those of *Δ113p53*^*M/M*^ mutant zebrafish. The *Δ113p53*^*M/M*^ mutant generated in our previous study exhibits relatively normal development and carries an 11-bp deletion in a p53 responsive element in the *Δ113p53* promoter located in the 4th intron of *p53*, which abolishes the expression of *Δ113p53* but does not influence the expression of full-length p53^44^. The results showed that there were no visible differences between uninjured *Δ113p53*^*M/M*^ mutant and WT hearts (Fig. S4), which suggests that the expression of *Δ113p53* in heart development is weak and that *Δ113p53* plays a small role in heart development. However, the percentage of the injury area containing the fibrin clot was significantly larger in *Δ113p53*^*M/M*^ mutant hearts (4.94%) (Fig. 2m, n) than in WT hearts (2.67%) (Fig. 2l, n) at 30 dpa. These results demonstrate that *Δ113p53* is induced to promote heart regeneration.

### *Δ113p53* has little effect on the activation of *gata4*-positive cardiomyocytes and their migration to the edge of the wound site

**Fig. 3:**
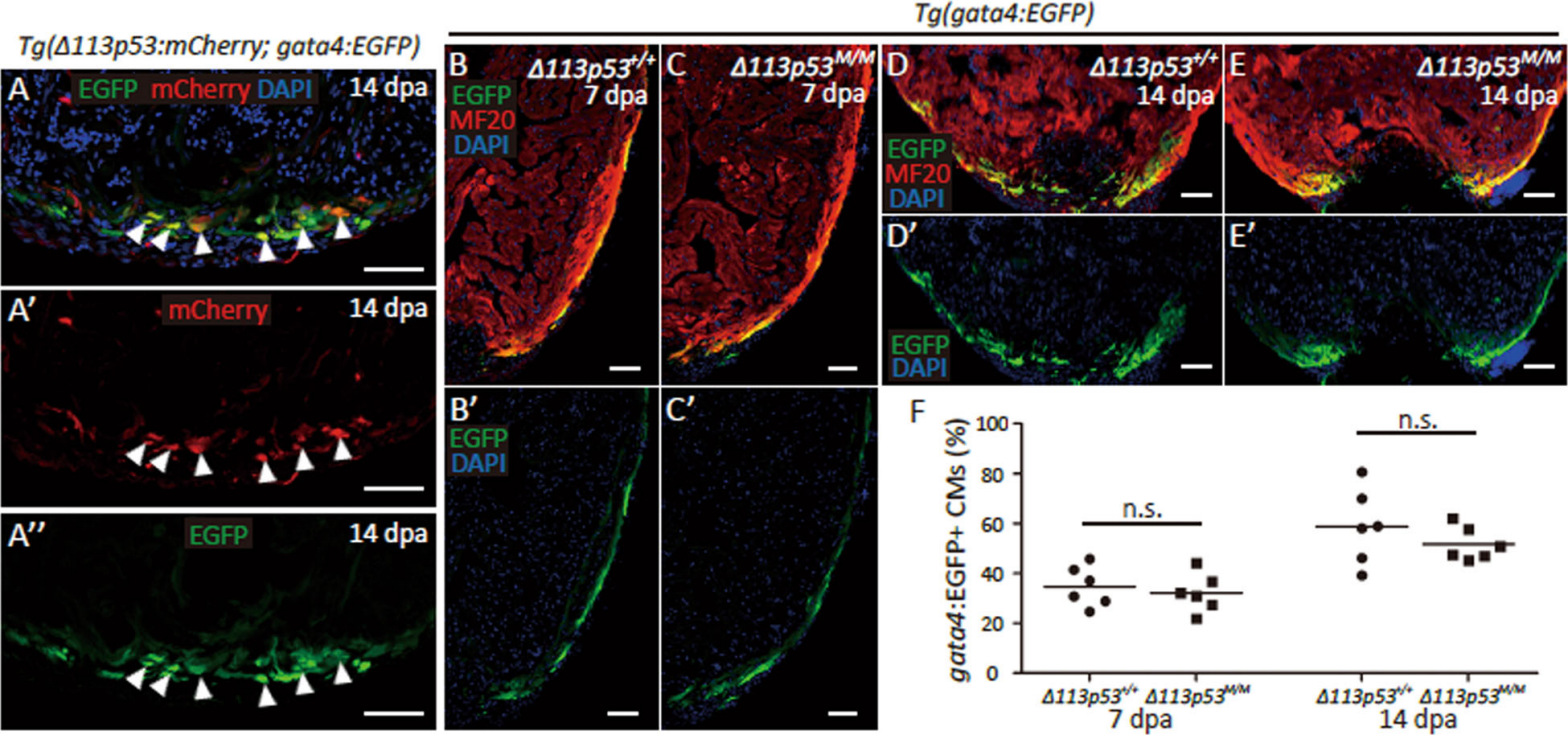
Depletion of Δ113p53 does not significantly impair the activation of *gata4*^+^ cells and their migration to the edge of the wound site during heart regeneration. **a** Co-localisation of red (*Δ113p53*^+^ cells) (**a**′) and green fluorescence (*gata4*^+^ cells) (**a**′′) on the cryosections of *Tg(Δ113p53:mCherry; gata4:EGFP)* hearts at 14 dpa. The white arrow heads indicate co-labelling. The nucleus were stained with DAPI (in blue). The representative picture was taken from three hearts. Scale bar, 50 μm. **b**–**e** Cryosections of *Tg(gata4:EGFP); Δ113p53*^*+/+*^ (**b**, **b**′, **d, d**′) and *Tg(gata4:EGFP); Δ113p53*^*M/M*^ hearts (**c**, **c**′, **e**, **e**′) at 7 and 14 dpa, were immunostained by anti-GFP (in green) and anti-MF20 (in red) antibodies. The nucleus were stained with DAPI (in blue). Scale bar, 50 μm. **f** Average size of EGFP^+^ cardiomyocytes on the edge of wound site in *Tg(gata4:EGFP); Δ113p53*^*+/+*^ and *Tg(gata4:EGFP); Δ113p53*^*M/M*^ mutant zebrafish at 7 and 14 dpa, was presented as the percentage of the ventricular area at the resection site. Data are means of three sections/heart from six hearts/time point. Scale bar, 50 μm. Each dot represents an individual heart. The experiments were repeated independently for at least three times with similar results. Statistical analysis was performed on relevant data using Student’s two-tailed *t* test in GraphPad Prism 5. The *p* values were represented by n.s. and asterisks. n.s., *p* > 0.05. *p < 0.05. **p < 0.01. ***p < 0.001.

The *gata4*-positive cardiomyocytes dedifferentiated from cardiomyocytes in the subepicardial ventricular layer migrate to the injury site and proliferate to contribute to zebrafish heart regeneration^5,6^. To investigate whether *Δ113p53*-positive cells were dedifferentiated cardiomyocytes, we generated *tg(Δ113p53:mCherry)* transgenic zebrafish by using a 3.6-kb fragment of the *Δ113p53* promoter to drive *mCherry* expression (Fig. S5) and crossed them with *Tg(gata4:EGFP)* zebrafish to obtain *tg(Δ113p53:mCherry*; *gata4*:*EGFP)* double transgenic fish. Immunostaining assays showed that mCherry was co-expressed with EGFP in some EGFP^+^ cardiomyocytes near the wound site at 14 dpa (Fig. 3a). These results demonstrate that *Δ113p53*^*+*^ cells are dedifferentiated cardiomyocytes.

Next, *Tg(gata4:EGFP)* transgenic reporter zebrafish were used to track newly regenerated cardiomyocytes in injured *Δ113p53*^*M/M*^ mutant hearts. We found that there were no visible differences in the location or percentage of *gata4*-positive cardiomyocytes in WT and *Δ113p53*^*M/M*^ mutant hearts at 7 dpa (Fig. 3b, c, f). Similar to those in the WT hearts, *gata4*-positive cardiomyocytes in *Δ113p53*^*M/M*^ mutant hearts migrated to the edge of the wound site at 14 dpa (Fig. 3d, e), although the percentage of *gata4*-positive cardiomyocytes at the edge of the wound site was slightly lower in *Δ113p53*^*M/M*^ mutant hearts than in WT hearts at 14 dpa (Fig. 3d–f). However, unlike in WT hearts, *gata4*-positive cardiomyocytes were rarely observed in the intermediate zone of the wound area in *Δ113p53*^*M/M*^ mutant hearts (Fig. 3d, e); it is unclear whether this phenomenon resulted from cardiomyocyte proliferation or from the penetration of *gata4*-positive cells. These results suggest that Δ113p53 does not play a critical role in cardiomyocyte dedifferentiation or the migration of *gata4*-positive cardiomyocytes from the outer compact layer of the ventricle to the edge of the wound site.

### *Δ113p53* promotes heart regeneration by enhancing cardiomyocyte proliferation, but not by inhibiting cardiomyocyte apoptosis

A recent study showed that cryoinjury triggers the DNA damage response during zebrafish heart regeneration^49^. Our previous studies revealed that Δ133p53 is induced during cell reprogramming to promote reprogramming efficiency through its anti-apoptotic activity and ensure the genomic integrity of induced pluripotent stem cells by increasing DNA DSB repair^50^. To compare apoptotic activity and the DNA damage response between the ventricles of WT and *Δ113p53*^*M/M*^ mutant hearts during regeneration, the *tg(myl7:nDsRed)* transgenic line (in which the promoter of zebrafish *myosin light chain 7* drives the expression of nuclear *DsRed*) was crossed onto the *Δ113p53*^*M/M*^ mutant background. The TUNEL assay and immunostaining for γ-H2AX (an early marker of the DNA damage response) were performed to analyse apoptotic cells and the DNA damage response, respectively. We found that there were only a few apoptotic cardiomyocytes and γ-H2AX-positive cardiomyocytes (co-stained with nDsRed) in the wound site in both WT and *Δ113p53*^*M/M*^ mutant hearts at 14 dpa (Fig. S6). These results suggest that 15% resection of the ventricle does not trigger a strong DNA damage response in cardiomyocytes during heart regeneration.

**Fig. 4:**
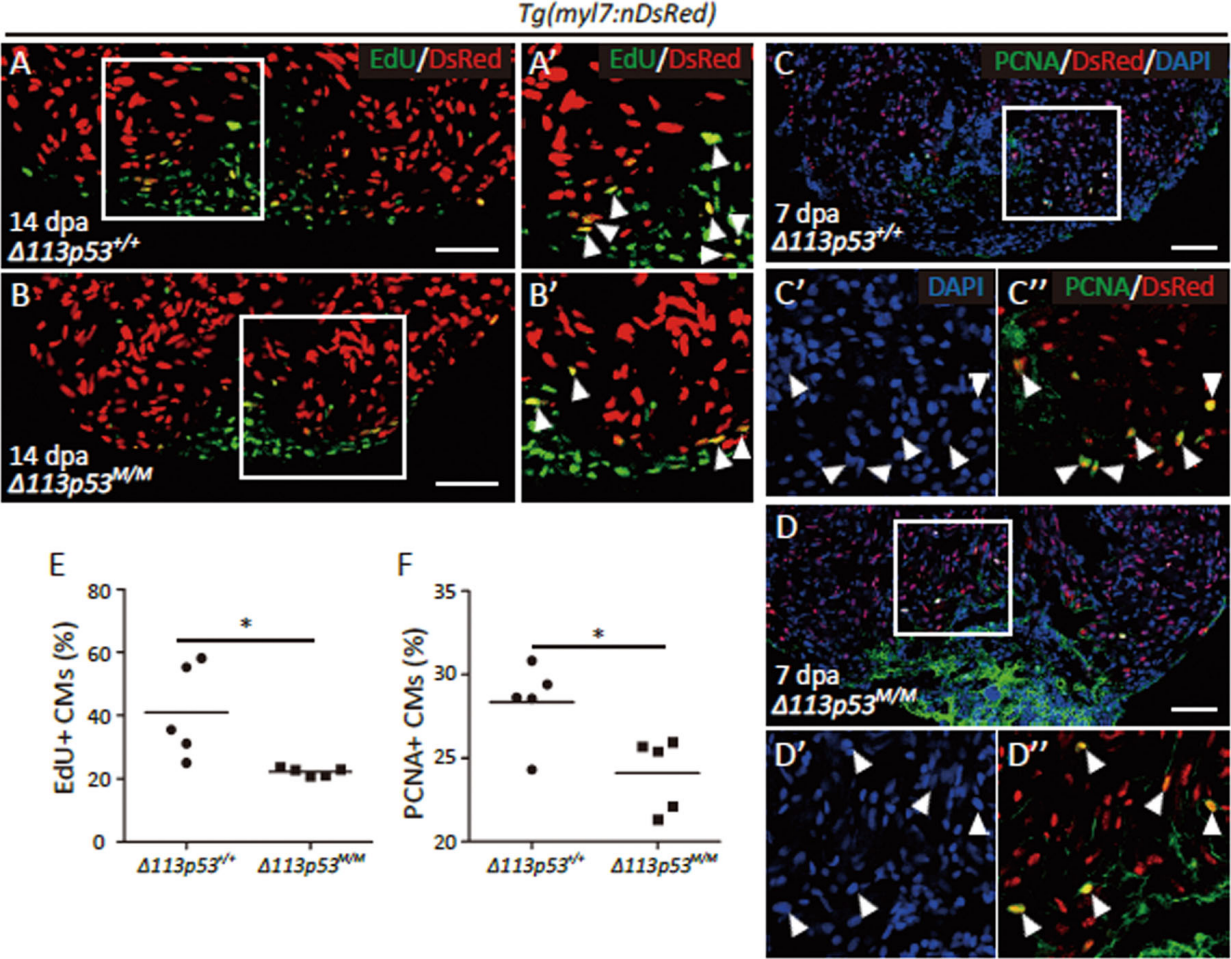
The depletion of *Δ113p53* significantly decreases the proliferation frequency of cardiomyocytes during heart regeneration. **a**, **b** The DsRed^+^ nucleus of cardiomyocytes (in red) at the resection site in *Tg(myl7:nDsRed); Δ113p53*^*+/+*^ **(a)** and *Tg(myl7:nDsRed); Δ113p53*^*M/M*^ (**b**) hearts were labelled by EdU (in green) at 14 dpa. Framed areas were magnified in **a**′ and **b**′. nDsRed: nuclear DsRed. The white arrow heads indicate co-labelling. Scale bar, 50 μm. **c**, **d** Cryosections of *Tg(myl7:nDsRed); Δ113p53*^*+/+*^ (**c**) and *Tg(myl7:nDsRed); Δ113p53*^*M/M*^ (**d**) hearts at 7 dpa were co-stained by anti-DsRed and anti-PCNA antibodies. The nucleus were stained with DAPI (in blue). Framed areas were magnified in **c′** and **c″**, or **d′** and **d″**. The white arrow heads indicate co-labelling. Scale bar, 50 μm. **e**, **f** The number of co-labelled nDsRed^+^ nucleus of cardiomyocytes with either EdU (**e**) or PCNA (**f**) in *Tg(myl7:nDsRed); Δ113p53*^*+/+*^ and *Tg(myl7:nDsRed); Δ113p53*^*M/M*^ hearts at 14 or 7 dpa, was presented as the percentage of the total nDsRed^+^ nucleus at the resection site. Data are means of three sections/heart. Each dot represents the average number of co-labelled nDsRed^+^ nucleus of cardiomyocytes with either EdU or PCNA in an individual heart. The experiments were repeated independently for at least three times with similar results. Statistical analysis was performed on relevant data using Student’s two-tailed *t* test in GraphPad Prism 5. The *p* values were represented by n.s. and asterisks. n.s., *p* > 0.05. *p < 0.05. **p < 0.01. ***p < 0.001.

To compare myocardial proliferation in the ventricles of WT and *Δ113p53*^*M/M*^ mutant hearts during regeneration, we quantified injury-induced cardiomyocyte proliferation by counting EdU^+^/Myl7^+^ or PCNA^+^ (the DNA replication marker proliferating cell nuclear antigen)/Myl7^+^ double-positive cardiomyocytes during heart regeneration. Compared to WT hearts, *Δ113p53*^*M/M*^ mutant hearts harboured significantly fewer proliferating cardiomyocytes labelled with EdU^+^/Myl7^+^ at 14 dpa (54% of the number in WT hearts) (Fig. 4a, b, e) and with PCNA^+^/Myl7^+^ at 7 dpa (85% of the number in WT hearts) (Fig. 4c, d, f). These data reveal that *Δ113p53* is required for cardiomyocyte proliferation following injury.

### *Δ113p53* upregulates the expression of antioxidant genes to maintain redox homeostasis during heart regeneration

**Fig. 5:**
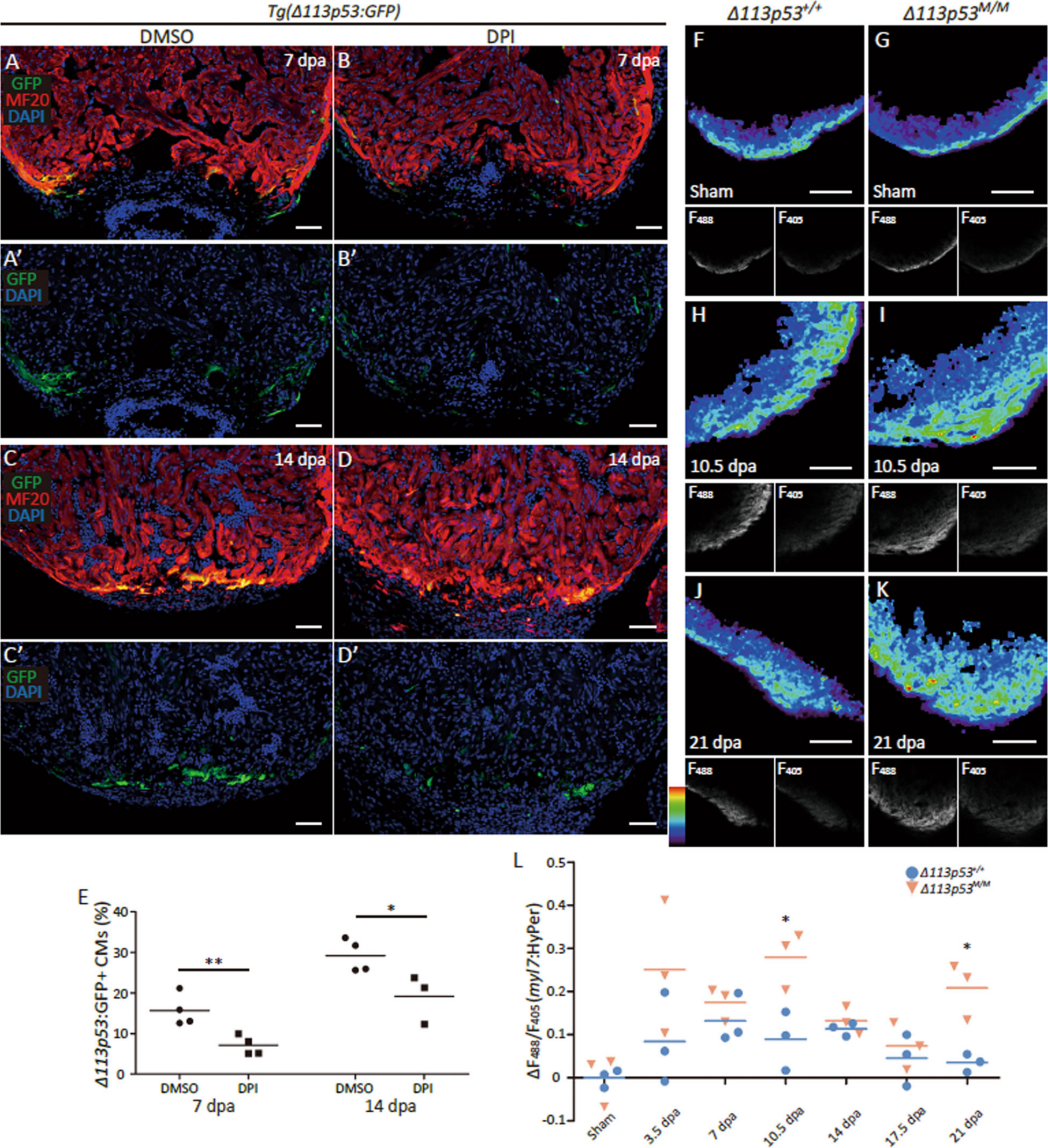
The increasing extents of H_2_O_2_ levels were significantly higher in the injury *Δ113p53*^*M/M*^ mutant hearts. (**a**–**d)** After surgery, *Tg(Δ113p53:GFP)* animals were treated with either DMSO (**a**, **a**′, **c**, **c**′) or DPI (**b**, **b**′, **d**, **d**′) daily at 3–7 or 7–14 dpa. The treated animals were sampled at 7 and 14 dpa and subjected to cryosection. Cryosections of hearts were immunostained by anti-GFP (in green) and anti-MHC (MF20) (in red) antibodies. The nuclei were stained with DAPI (in blue). Scale bar, 50 μm. **e** Average size of GFP^+^ cardiomyocytes on heart sections of *Tg(Δ113p53:GFP)* treated with DMSO or DPI at 7 and 14 dpa, was presented as the percentage of the ventricular area at the resection site. Data are means of three sections/heart. Each dot represents the average size of GFP^+^ cardiomyocytes in an individual heart. Scale bar, 50 μm. **f**–**l** Ex vivo HyPer heart images of either *Δ113p53*^*+/+*^ (**f**, **h**, **j**) or *Δ113p53*^*M/M*^ mutant hearts (**g**, **i**, **k**) at sham, 10.5 and 21 dpa. Spatially resolved H_2_O_2_ image, indexed by the ratio between the *F*_488_ and *F*_405_ images of HyPer (below), is presented in pseudocolor. Ratiometric HyPer signals (*F*488/*F*405) averaged over the regenerative zone of injured heart at 3.5, 7, 10.5, 14, 17.5 and 21 dpa were presented as the difference to the average *F*_488_*/F*_405_ ratio at the apex of respective sham hearts (**l**). Each dot represents the ratiometric HyPer signal in an individual heart. Statistical analyses were performed on data from *Δ113p53*^*+/+*^ and *Δ113p53*^*M/M*^ mutant hearts at the same time point. The experiments were repeated independently for at least three times with similar results. Statistical analysis was performed on relevant data using Student’s two-tailed *t* test in GraphPad Prism 5. The *p* values were represented by n.s. and asterisks. n.s., *p* > 0.05. *p < 0.05. **p < 0.01. ***p < 0.001.

A recent study revealed that H_2_O_2_ is produced near the wound site of ventricles to promote heart regeneration^16^. Our previous study demonstrated that the human orthologue Δ133p53 is induced in response to sub-toxic levels of ROS to promote cell proliferation by upregulating the expression of antioxidant genes^46^. Therefore, we investigated whether the induction of *Δ113p53* is related to maintaining redox homeostasis during heart regeneration. For this purpose, we treated *tg(Δ113p53:GFP)* zebrafish with diphenylene iodonium (DPI), an NADPH oxidase (Duox/Nox enzymes) inhibitor, to block the production of H_2_O_2_^16^ after amputation. The results showed that compared to control treatment, DPI treatment significantly reduced the percentage of *Δ113p53*^*+*^ cardiomyocytes near the wound site at 7 and 14 dpa (Fig. 5a–e), suggesting that the induction of *Δ113p53* depends on elevation of ROS levels during heart regeneration.

Next, we determined the status of ROS in the injured hearts of both WT and *Δ113p53*^*M/M*^ mutant zebrafish at different time points with the *tg(myl7:HyPer)* transgene (in which the promoter of *myl7* drives the expression of HyPer, a fluorescent protein-based H_2_O_2_ sensor)^16^. Similar to a previous study^16^, the level of H_2_O_2_ in the injured WT hearts started to increase at 3 dpa, decreased beginning at 14 dpa and reached the basal level at 21 dpa (Fig. 5f, h, j, l), whereas the H_2_O_2_ levels in the injured *Δ113p53*^*M/M*^ mutant hearts were significantly higher than those in the injured WT hearts at 10.5 and 21 dpa (Fig. 5g, i, k, l). These results suggest that depletion of *Δ113p53* results in elevated levels of intracellular H_2_O_2_ during heart regeneration.

**Fig. 6:**
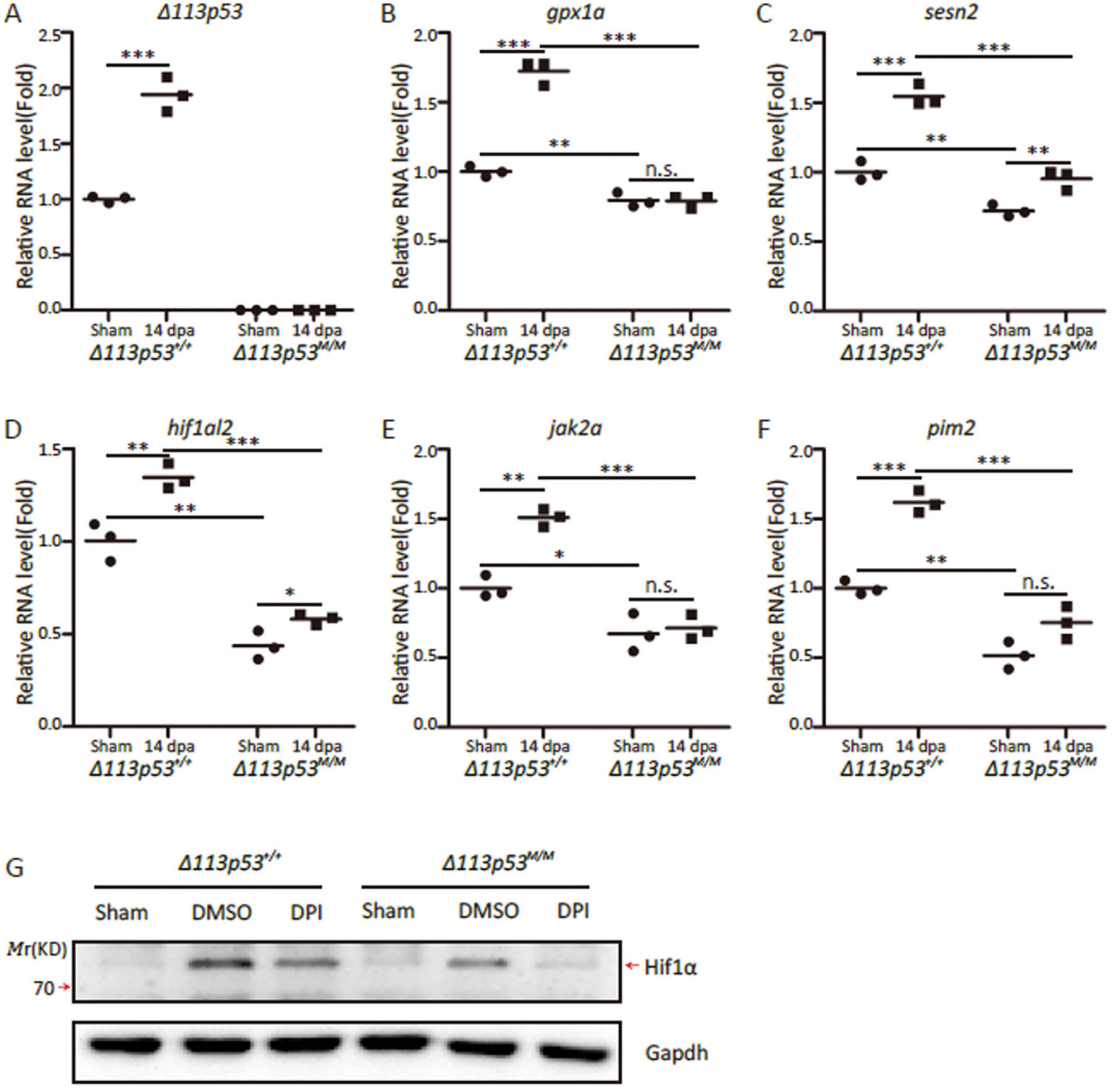
Upregulation of antioxidant and proproliferative genes during heart regeneration is impaired in the injury *Δ113p53*^*M/M*^ mutant hearts. **a**–**f** Relative mRNA expression of *Δ113p53* (**a**), *gpx1a* (**b**), *sesn2* (**c**), *hif1al2* (**d**), *jak2a* (**e**) and *pim2* (**f**) in the *Δ113p53*^*+/+*^ and *Δ113p53*^*M/M*^ hearts at sham and 14 dpa. The total RNA was extracted from a pool of at least 10 hearts in each group. **g** Western blot was performed to analyse the induction of zebrafish Hif1α in different samples as indicated. The *Δ113p53*^*+/+*^ and *Δ113p53*^*M/M*^ mutant zebrafish with heart resection were treated with DPI from 3 to 7 dpa. Total protein was isolated from four hearts/treatment at 7 dpa and subjected to western blot analysis. Gapdh was used as the protein loading control. The experiments were repeated independently for at least three times with similar results. Statistical analysis was performed on relevant data using Student’s two-tailed *t* test in GraphPad Prism 5. The *p* values were represented by n.s. and asterisks. n.s., *p* > 0.05. *p < 0.05. **p < 0.01. ***p < 0.001.

To investigate whether elevated ROS levels in the injured *Δ113p53*^*M/M*^ mutant hearts were related to antioxidant genes, we examined the expression of six antioxidant genes (p53 target genes), including *gpx1a*, *sesn2*, *aldh4*, *sesn1*, *sod1* and *sod2*, by using qRT-PCR. The expression of *gpx1a* and *sesn2*, as well as the expression of *Δ113p53* was significantly upregulated in the injured WT hearts (Fig. 6a–c) compared to the sham hearts at 14 dpa, whereas the expression of the remaining 4 genes was not significantly changed (Fig. S7). Interestingly, the expression of all six antioxidant genes in sham *Δ113p53*^*M/M*^ mutant hearts was lower than that in sham WT hearts (Figs. 6b, c andS7). Furthermore, the induction of *gpx1a* was not triggered in injured *Δ113p53*^*M/M*^ mutant hearts at 14 dpa (Fig. 6b), while the induction of *sesn2* was significantly lower in injured *Δ113p53*^*M/M*^ mutant hearts than in injured WT hearts at 14 dpa; however, the expression of *sesn2* was increased in injured *Δ113p53*^*M/M*^ mutant hearts compared to sham *Δ113p53*^*M/M*^ mutant hearts (Fig. 6c). These results demonstrate that the antioxidant response is triggered in wounded hearts and that *Δ113p53* promotes the expression of antioxidant genes. This result also implies that the elevation of ROS levels in the injured *Δ113p53*^*M/M*^ mutant hearts is due to lower expression of antioxidant genes.

Finally, we tried to gain insight into the role of elevated ROS levels in cardiomyocyte proliferation. ROS stress elicits the ATM-homodimer-Chk2 pathway to trigger the DNA damage response^51^. However, our results showed that apoptotic activity and the DNA damage response was rarely induced by 15% ventricular resection in both WT and *Δ113p53*^*M/M*^ mutant hearts (Fig. S6). A previous study revealed that cardiac injury induces the hypoxia response in zebrafish ventricles, resulting in activation of Hif1α signalling, which promotes cardiomyocyte proliferation by upregulating the expression of numerous proproliferative genes, including many components of the Jak-STAT pathway^52^. A number of studies have also documented that an increase in ROS levels can downregulate Hif1α signalling^53–55^. Therefore, we evaluated the expression of three genes in the Hif1α signalling pathway, including *hif1al2* (*hypoxia inducible factor 1 subunit alpha, like 2*), *jak2a* and *pim2* (two HIF1α downstream genes), by qRT-PCR. Consistent with a previous study^52^, the expression of these three genes was upregulated in injured WT hearts (Fig. 6d–f) compared to sham hearts at 14 dpa. The expression of all three genes in sham *Δ113p53*^*M/M*^ mutant hearts was lower than that in sham WT hearts (Fig. 6d–f). Although the expression of *hif1al2* was also upregulated in injured *Δ113p53*^*M/M*^ mutant hearts at 14 dpa (Fig. 6d), the induction of two downstream genes, *jak2a* and *pim2*, was abolished in injured *Δ113p53*^*M/M*^ mutant hearts at 14 dpa.(Fig. 6e, f). These results suggest that the elevation of ROS levels may repress cardiomyocyte proliferation through inactivating the Hif1α signalling pathway.

To verify the activation of *hif1α* in heart regeneration and address if the *hif1α* activation is dependent on the ROS signal, we treated WT and *Δ113p53*^*M/M*^ mutant zebrafish with DPI to block the production of H_2_O_2_ after amputation and analysed the level of Hif1α protein at 7 dpa. The western blot analysis confirmed that the expression of Hif1α was induced in both WT and *Δ113p53*^*M/M*^ mutant resected hearts, compared to that in respective sham hearts (Fig. 6g). Interestingly, DPI treatment observably reduced the activation of Hif1α protein in both WT and *Δ113p53*^*M/M*^ mutant resected hearts at 7 dpa (Fig. 6g), suggesting that the induction of Hif1α depends on elevation of ROS levels during heart regeneration.

## Discussion

It is well documented that ROS are produced after tissue injury and play an important role in wound healing by initiating acute inflammation, clarifying infection and dead tissue, and mediating various intracellular signal transduction pathways^56–58^. However, when the level of ROS is beyond a cell’s capacity to maintain redox homeostasis, oxidative stress occurs, which results in direct or indirect ROS-mediated damage to nucleic acids, proteins and lipids^20,21^. Therefore, ROS levels in cells are tightly controlled by antioxidant systems^22^. P53 and its isoform Δ133p53/Δ113p53 play a critical role in the maintenance of redox homeostasis by regulating the expression of antioxidant genes^46^. Interestingly, ROS are also generated during zebrafish heart regeneration to promote cardiomyocyte proliferation^16^, and the p53 signalling pathway is activated during cardiac regeneration in neonatal mice^47^. However, how redox homeostasis is maintained and whether p53 signalling plays a role in heart regeneration remain unclear.

In this report, we applied partial zebrafish ventricular resection to investigate the function of *Δ113p53* in heart regeneration. Based on a p53-based genetic tracing system involving the insertion of a CreER cassette immediately after the first ATG of the full-length mouse p53 BAC clone (located in the second exon of p53), a previous study revealed that full-length *p53*-positive cardiomyocytes are activated by injury in neonatal mice and undergo proliferation to contribute to heart regeneration^47^. In contrast, using *Δ113p53* transgenic reporter fish, in situ hybridisation and qRT-PCR, we found that the transcription of *Δ113p53*, but not full-length p53, was induced in cardiomyocytes near the injury site in zebrafish ventricles (Fig. 1a–j). The induction of *Δ113p53* was not observed in injured *p53*^*M214K*^ mutant hearts (Fig. 1k, l), which is consistent with *Δ113p53* being a p53 target gene^40^. The discrepancy between the two studies in mouse and zebrafish may be explained by the fact that the mouse p53 reporter system contains the first exon of p53^47^, which might be the promoter for the mouse *Δ113p53/Δ133p53* orthologue. Next, we explored the function of *Δ113p53* in heart regeneration in *Δ113p53*^*M/M*^ mutants. Masson’s staining showed that the area of the injury containing the fibrin clot was significantly increased in the wound site in *Δ113p53*^*M/M*^ mutant hearts (Fig. 2l–n) compared to WT hearts at 30 dpa, which demonstrates that heart regeneration is impaired in the *Δ113p53*^*M/M*^ mutants. Although there were no observable differences in dedifferentiation to *gata4*-positive cardiomyocytes (Fig. 3b–f) or cardiomyocyte apoptosis between injured WT and *Δ113p53*^*M/M*^ mutant hearts (Fig. S6c, d), the percentages of EDU-labelled cardiomyocytes and PCNA-labelled cardiomyocytes were significantly lower in injured *Δ113p53*^*M/M*^ mutant hearts than in injured WT hearts (Fig. 4). These results reveal that *Δ113p53* promotes heart regeneration by increasing cardiomyocyte proliferation. Further analysis showed that H_2_O_2_ levels in the injured *Δ113p53*^*M/M*^ mutant hearts were significantly higher than those in the injured WT hearts (Fig. 5f–l) and that the increase in H_2_O_2_ levels was coincident with a decrease in antioxidant gene expression in the injured *Δ113p53*^*M/M*^ mutant hearts (Fig. 6b, c). These results suggest that Δ113p53 promotes cardiomyocyte proliferation by maintaining redox homeostasis.

Taken together, our findings demonstrate that although ROS signalling plays an important role in promoting heart regeneration^16^, the level of ROS should be tightly controlled. The induction of Δ113p53 functions to maintain redox homeostasis by promoting antioxidant gene expression.

Oxidative stress has been implicated in human cardiac diseases, including ischaemia-reperfusion (IR), myocardial infarction (MI) and heart failure^57,59^. ROS are produced in two stages, namely, ischaemia and reperfusion, at low and high levels, respectively^60^. ROS play a dual role in tissue injuries, as massive amounts of mitochondrial ROS induce apoptosis and necrosis of cells^61^, whereas moderate levels of ROS promote cell survival and proliferation^16,62,63^. Therefore, maintaining redox homeostasis plays an important role in the mechanisms of and therapeutic strategies for cardiac diseases. It has also been reported that during pressure overload, the activation of full-length p53 has a crucial function in the transition from cardiac hypertrophy to heart failure by repressing Hif1 activity^64^. Here, we demonstrate that Δ113p53 is induced by ROS during zebrafish heart regeneration and functions to promote cardiomyocyte proliferation by maintaining redox homeostasis and Hif1α activity. Our results suggest that the expression of Δ133p53 may also be activated during IR and protect patients from IR-induced heart failure.

## Methods and materials

### Zebrafish lines

Zebrafish were raised and maintained in standard zebrafish units at Zhejiang University as described previously^44^. The *Tg(Δ113p53:GFP)* transgenic line and *Δ113p53*^*M/M*^ mutant zebrafish were generated in our previous studies^40,44^. A 3.6-kb fragment of the *Δ113p53* promoter^40^ was used to create the *Tg(Δ113p53:CreER)* and *tg(Δ113p53:mCherry)* transgenic lines on the AB genetic background through Tol2-based transgenesis^40^. *p53*^*M214K*^ mutant^48^, *Tg(β-act2:RSG)*^5^, *Tg(gata4:EGFP)*^65^, *Tg(myl7:nDsRed)*^66^ and *Tg(myl7:HyPer)*^16^ zebrafish were generated by different labs as previously reported.

### Ethics statement

All animal procedures were performed in full accordance with the requirements of the Regulation for the Use of Experimental Animals of Zhejiang Province. This work was specifically approved by the Animal Ethics Committee of the School of Medicine, Zhejiang University (ethics code permit no. ZJU20190012).

### Adult zebrafish heart resection

Ventricular surgery was performed on 5- to 10-month-old zebrafish according to previously described procedures^2^. Briefly, zebrafish were anaesthetised with 0.02% Tricaine and then subjected to ~15% ventricular amputation at the apex with scissors.

### Quantitative real-time reverse transcriptional PCR

Hearts were freshly isolated from anaesthetised zebrafish subjected to sham surgery or resection at different time points. The outflow tracts and atriums were removed from the isolated hearts. Total RNA was extracted from ~10 isolated ventricles from each group using a homogeniser (JXFSTPRP-24, Shanghai Jingxin) in Invitrogen TRIzol reagent (Cat No. 15596026). Isolated RNA was treated with DNaseI (NEB, M0303S) prior to reverse transcription and purified through lithium chloride. First-strand cDNA was synthesised using M-MLV Reverse Transcriptase (Invitrogen, C28025021). The reaction was performed using a CFX96^TM^ Real-Time System (Bio-Rad) with AceQ qPCR SYBR Green (Vazyme, Q111-02) according to the manufacturer’s instructions. Total RNA levels were normalised to the level of β-actin. Statistics were obtained from three repeats. The primer sequences of the analysed genes are listed in Table S1.

### In situ hybridisation

For the in situ hybridisation assay, isolated zebrafish hearts were fixed in 4% PFA for 2 days before cryosectioned. The probes were generated by NEB T7 RNA Polymerase (M0251S) and Roche DIG RNA Labelling Mix (11277073910) from a *Δ113p53-pCS2*^*+*^ plasmid constructed in our previous study^44^. Staining was performed with Anti-Digoxigenin-AP (Roche, 11093274910) and the BCIP/NBT Alkaline Phosphatase Colour Development Kit (Beyotime Biotechnology, C3206).

### Ex vivo intact heart imaging

Ex vivo *Tg(myl7:HyPer)* heart imaging and image processing were performed according to previously described procedures^16^. Briefly, images were taken under an Olympus FV1000 upright confocal microscope, and the HyPer 488/405 ratio was calculated based on the integrated optic density using Adobe Photoshop CS5.

### EdU incorporation assay and small-molecule treatment

For the EdU incorporation assay, 15 μL of 100 mM EdU (Invitrogen, A10044) was injected once daily into the abdominal cavity of each animal that underwent surgery for 3 or 7 days until the hearts were collected at 7 or 14 dpa. The hearts were then fixed for cryosectioning. EdU staining was performed using Azide Alexa Fluor 647 (Invitrogen, A10277).

For DPI treatment, 50 μL of 10 μM DPI (Sigma, D2926) was injected daily into the thoracic cavity of each animal that underwent surgery beginning 3 or 7 dpa until the hearts were collected at 7 or 14 dpa^16^.

For the cell lineage tracing assay, *tg(Δ113p53:CreER; β-act2:RSG)* fish subjected to sham surgery or surgery were bathed in 3 μM 4HT (Sigma, H7904) for 24 h at 7 and 10 dpa as previously described^15^.

### Western blot, immunostaining and histological methods

For the western blot assay, a zebrafish p53 monoclonal antibody was generated by HuaAn Biotechnology (Hangzhou, China) as previously described^67^. A human HIF1α antibody (BOSTER, A00013-1) was used to detect zebrafish Hif1a. A β-actin antibody (Huabio, R1207-1) was used as the protein loading control for the experiments in embryonic stages. A Gapdh antibody (HuaBio, R1208-3) was used as the protein loading control for the experiments in zebrafish heart regeneration. The secondary antibodies were HRP-conjugated goat anti-mouse IgG (Huabio, HA1006) and HRP-conjugated goat anti-rabbit IgG (Huabio, HA1001).

Zebrafish hearts were fixed, cryosectioned (14 μm) as described previously^2^ and then subjected to immunostaining. The primary antibodies were anti-GFP (Abcam, ab13970), anti-MYH1E (MF20; Developmental Studies Hybridoma Bank, AB 2147781), anti-PCNA (Sigma, P8825), anti-DsRed (Clontech, 632496) and anti-H2A.XS139ph (Genetex, GTX127340). The secondary antibodies were Alexa Fluor 488-conjugated anti-chicken IgY H&L (Abcam, ab150169), Alexa Fluor 647-conjugated anti-mouse IgG H&L (Abcam, ab150115), Dylight 549-conjugated anti-rabbit IgG H&L (EarthOx, E032320) and Alexa Fluor 647-conjugated anti-rabbit IgG H&L (Abcam, ab150143). Nuclei were stained with DAPI (BYT, C1002).

Masson’s staining was performed on cryosections using trichrome Masson’s staining solution from Servicebio (G1006). Myosin was visualised as red, and fibrin was visualised as blue. Images were captured under an Olympus BX53 microscope with a camera from Qimaging MicroPublisher 5.0 RTV.

### TUNEL assay

The TUNEL assay was performed on freshly prepared cryosections of *tg(myl7:nDsRed)* zebrafish hearts using a fluorescein-based Roche In Situ Cell Death Detection Kit (Cat No. 11684795910).

### Quantification and statistical analysis

Sample sizes were designed based on routine genetic analysis in zebrafish studies. The investigators were blinded to group allocation during data collection and analysis. No data were excluded from the analyses. All samples were randomly selected.

## Supplementary information

Supplementary Information

Supplementary Information

Supplementary Information

Supplementary Information

Supplementary Information

Supplementary Information

Supplementary Information

Supplementary Information
