# Supplementary material for: p53 isoform Δ113p53 promotes zebrafish heart regeneration by maintaining redox homeostasis
Source: Cell Death Dis. 2020 Jul 23;11(7):568. doi: 10.1038/s41419-020-02781-7 (PMC7378207; doi:10.1038/s41419-020-02781-7)
Supplement: Supplementary file 1 — Supplementary Information [file 41419_2020_2781_MOESM1_ESM.docx]

**Supplementary figure legends**

**Figure S1. *Δ113p53:*GFP was not detectable in the surgery hearts of *p53^M214K^* mutant fish at 14 dpa.** **(A-B)** Cryosections of *Tg(Δ113p53:GFP)* hearts of p53^+/+^ **(A)** and p53^M214K^ mutant **(B)** at 14 dpa were immunostained by anti-GFP (in green) and anti-MHC (MF20) (in red) antibodies. The nucleus were stained with DAPI (in blue). The representative picture was taken from 3 hearts in each group. The white arrow heads indicate wounding site. Scale bar, 50 μm.

**Figure S2.** **Statistical analysis of average numbers of Edu^+^/GFP^+^/MF20^+^ cells among the total of Edu^+^/MF20^+^ cells in *Tg(Δ113p53:GFP)* hearts at sham and 7 dpa.** The number of Edu^+^/GFP^+^/MF20^+^ cells on heart sections of *Tg(Δ113p53:GFP)* at sham and 7 dpa, as shown in **Figure 2A-C**, was presented as the percentage of the total Edu^+^/MF20^+^ cells at the wound site. Data are means of 4-6 sections/heart with the largest wound area from 3-7 hearts in different treatments. Scale bar, 50 μm. Each dot represents an individual heart.

**Figure S3. Validation of *Tg(Δ113p53:CreER; β-act2:RSG)* zebrafish.** **(A)** Western blot with a monoclonal antibody against zebrafish Δ113p53 was performed to analyze the induction of *Δ113p53* upon the treatment of a DNA damage drug, camptothecin (Campt). The *Tg(Δ113p53:CreER; β-act2:RSG)* zebrafish embryos at 1 day post fertilization (dpf) were treated with Campt for 24 hours. Afterwards, a part of untreated and Campt-treated embryos were divided and treated with 4HT for 2 hours. β-actin was used as the protein loading control. **(B-E)** Life images of red (DsRed) **(B, C, D, E)**, green (EGFP) **(B’, C’, D’, E’)** and bright field (**B”, C”, D”, E”**) in *Tg(Δ113p53:CreER; β-act2:RSG)* zebrafish embryos treated with either Campt **(C, C’, C”)**, or 4HT **(D, D’, D”)**, or both **(E, E’, E”)** as described above. Scale bar, 500 μm.

**Figure S4. *Δ113p53^M/M^* mutant and WT uninjured hearts are comparative. (A-B)** Trichrome Masson’s staining on the crysections of uninjured Δ113p53^+/+^ **(A)** and Δ113p53^M/M^ mutant hearts **(B)** at 9 months of age. The representative picture was taken from 5 hearts in each group. Scale bar, 50 μm.

**Figure S5. Validation of *Tg(Δ113p53:mCherry)* zebrafish.** (**A**) Diagram showing the *Tg(Δ113p53:mCherry)* reporter driven by *Δ113p53* promoter. *Δ113p53*-P (blue arrow): the 3.6 kb DNA fragment from the upstream of *Δ113p53* transcription start site; *mCherry* (red bar): the coding region of *mCherry*. **(B-C)** Live images of red (mCherry) fluorescence in *Tg(Δ113p53:mCherry)* zebrafish embryos treated with Campt **(C)** or without Campt **(B)** for 24 hours. Scale bar, 500 μm.

**Figure S6.** **Depletion of Δ113p53 has little effects on DNA damage response and apoptotic activity during heart regeneration. (A-B)** Cryosections of *Tg(myl7:nDsRed); Δ113p53^+/+^* **(A)** and *Tg(myl7:nDsRed); Δ113p53^M/M^* **(B)** hearts at 14 dpa were co-stained by anti-DsRed (in red) and anti-γ-H2AX (in green) antibodies. Framed areas were magnified in **A’** and **B’**. **(C-D)** TUNEL assay (in green) and co-immunostaining with anti-DsRed antibody (in red) were performed on cryosections of *Tg(myl7:nDsRed); Δ113p53^+/+^* **(C)** and *Tg(myl7:nDsRed); Δ113p53^M/M^* **(D)** hearts at 14 dpa. Framed areas were magnified in **C’** and **D’**. The representative picture was taken from 6 hearts in each group. Scale bar, 50 μm.

**Figure S7.** **(A-D) Relative mRNA expression of antioxidant genes:** *aldh4* **(A)**, *sesn1* **(B)**, *sod1* **(C)** and sod2 **(D)** in the Δ113p53^+/+^ and Δ113p53^M/M^ injury at sham and 14 dpa. The total RNA was extracted from a pool of at least 10 hearts in each group. Statistical analysis was performed on relevant data using Student’s two-tailed t-test in GraphPad Prism 5. The p values were represented by n.s. and asterisks. n.s., p > 0.05. *, p < 0.05. **, p < 0.01. ***, p < 0.001.

**Supplementary tables**

**Table. S1. The primer sequences and accession numbers of the analyzed genes in qRT-PCR**.

| Genes  (Accession) | Primer sequences | |
| --- | --- | --- |
|  | Forward | Reverse |
| *β-actin*  (NM_131031) | CATTGGCAATGAGCGTTTC | TACTCCTGCTTGCTGATCCAC |
| *p53*  (NM_001271820) | TGGAGAGGAGGTCGGCAAAATCAA | GACTGCGGGAACCTGAGCCTAAAT |
| *Δ113p53*  (NM_001328588) | ATATCCTGGCGAACATTTGGAGGG | CCTCCTGGTCTTGTAATGTCAC |
| *p21*  (NM_001128420) | TCCCGCATGAAGTGGAGAAA | GACGCTTCTTGGCTTGGTAG |
| *aldh4*  (NM_201158) | GAGCCCACAATAATCGAGAC | CAGATTTGTCTTGGGGGAAG |
| *gpx1a*  (NM_001007281) | GCACCAGGAGAACTGCAAG | TTCAGGAACGCAAACAGAGG |
| *sesn1*  (NM_001002660) | GGGTGTGGACACTTCCATGC | CCGGAACTGTCTCCAGAAGC |
| *sesn2*  (NM_001079975) | ATGTTCAGCGCTCCGTCCTT | CATCCAGAAGCTGCGCCATC |
| *sod1*  (NM_131294) | TGGGTAATGTGACCGCTGAT | ACTTTCCTCATTGCCACCCT |
| *sod2*  (NM_199976) | ACTATGGTGCACTTGAGCCT | CGCCATTGGGTGACAGATTT |
| *hif1al2*  (NM_001012371) | GATCGGAAGGAGCTTGCTTG | GTCATGTCAGGGCTGTGAAC |
| *jak2a*  (NM_131093) | GGAGACTTCGGCCTGACTAA | ACTGCAGCTCTTCTCACTGT |
| *pim2*  (NM_131539) | ATTGTTGACGTGCGTTTGGA | TGGGAACCGGGTTTACTTCA |
