# Supplementary figures and images for: p53 isoform Δ113p53 promotes zebrafish heart regeneration by maintaining redox homeostasis
Source: Cell Death Dis. 2020 Jul 23;11(7):568. doi: 10.1038/s41419-020-02781-7 (PMC7378207; doi:10.1038/s41419-020-02781-7)

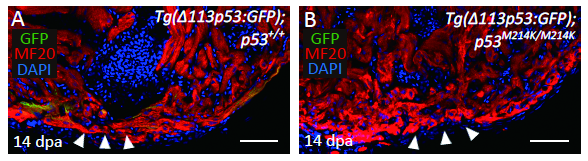

Supplement: Supplementary file 2 — Supplementary Information [file 41419_2020_2781_MOESM2_ESM.tif]

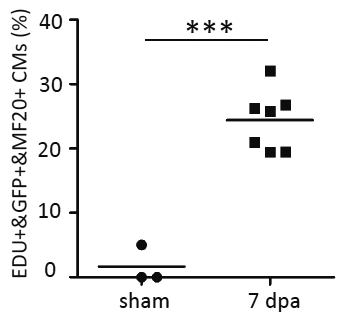

Supplement: Supplementary file 3 — Supplementary Information [file 41419_2020_2781_MOESM3_ESM.tif]

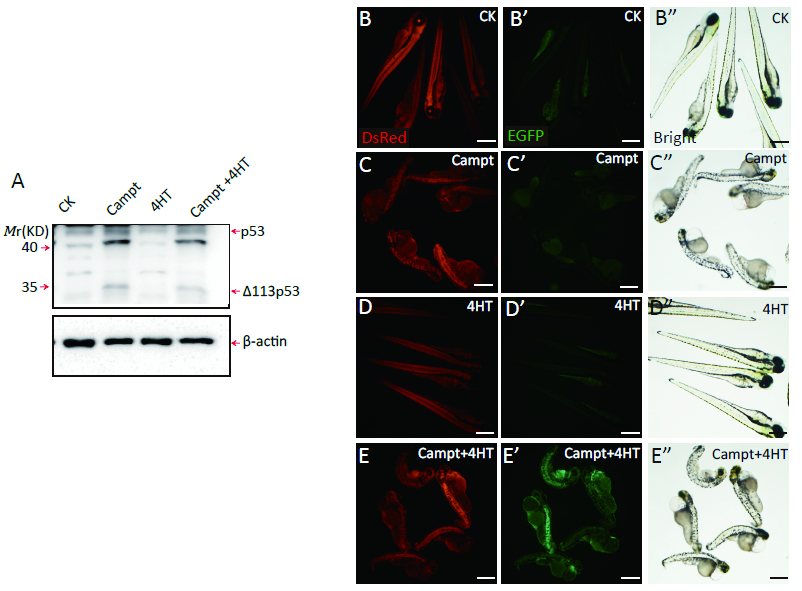

Supplement: Supplementary file 4 — Supplementary Information [file 41419_2020_2781_MOESM4_ESM.tif]

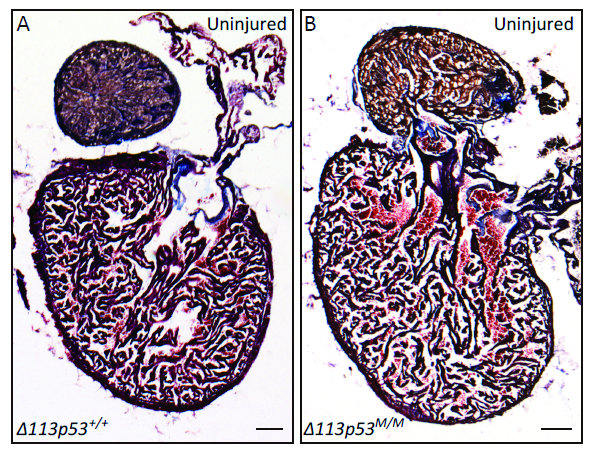

Supplement: Supplementary file 5 — Supplementary Information [file 41419_2020_2781_MOESM5_ESM.tif]

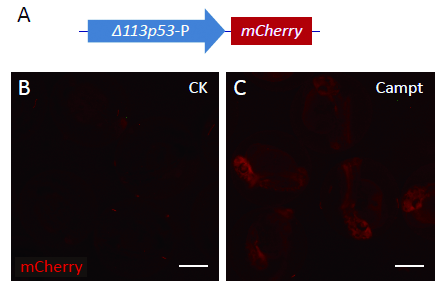

Supplement: Supplementary file 6 — Supplementary Information [file 41419_2020_2781_MOESM6_ESM.tif]

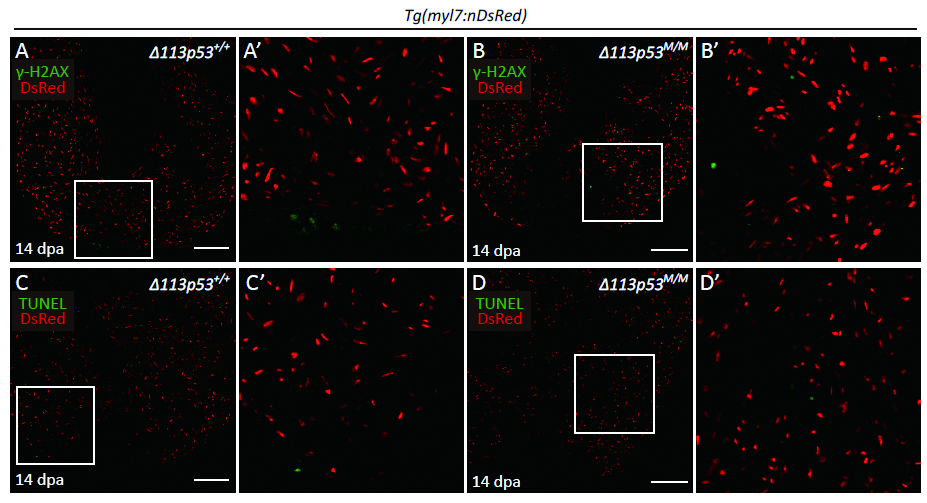

Supplement: Supplementary file 7 — Supplementary Information [file 41419_2020_2781_MOESM7_ESM.tif]

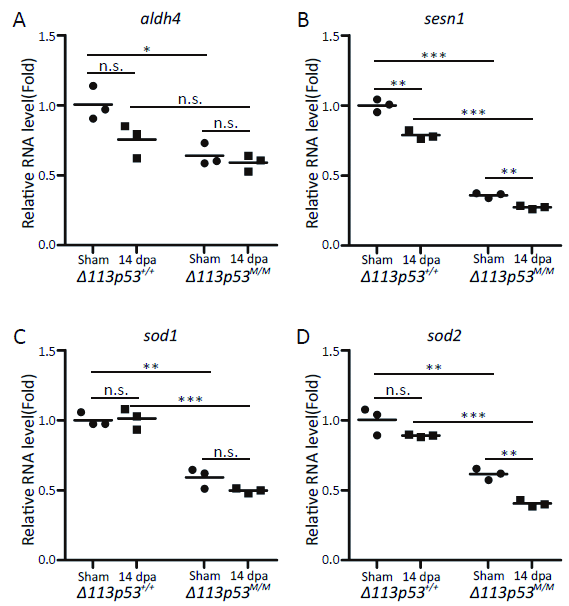

Supplement: Supplementary file 8 — Supplementary Information [file 41419_2020_2781_MOESM8_ESM.tif]
